# An integrated approach to reveal miRNAs’ impacts on the functional consequence of copy number alterations in cancer

**DOI:** 10.1038/srep11567

**Published:** 2015-06-23

**Authors:** Kening Li, Yongjing Liu, Yuanshuai Zhou, Rui Zhang, Ning Zhao, Zichuang Yan, Qiang Zhang, Shujuan Zhang, Fujun Qiu, Yan Xu

**Affiliations:** 1 College of Bioinformatics Science and Technology, Harbin Medical University, Harbin 150081, China; 2 School of Life Sciences and Biotechnology, Shanghai Jiao Tong University, Shanghai 200240, China

## Abstract

Copy number alteration (CNA) is known to induce gene expression changes mainly through dosage effect, and therefore affect the initiation and progression of tumor. However, tumor samples exhibit heterogeneity in gene dosage sensitivity due to the complicated mechanisms of transcriptional regulation. Currently, no high-throughput method has been available for identifying the regulatory factors affecting the functional consequences of CNA, and determining their effects on cancer. In view of the important regulatory role of miRNA, we investigated the influence of miRNAs on the dosage sensitivities of genes within the CNA regions. By integrating copy number, mRNA expression, miRNA expression profiles of three kinds of cancer, we observed a tendency for high dosage-sensitivity genes to be more targeted by miRNAs in cancer, and identified the miRNAs regulating the dosage sensitivity of amplified/deleted target genes. The results show that miRNAs can modulate oncogenic biological functions by regulating the genes within the CNA regions, and thus play a role as a trigger or balancer in cancer, affecting cancer processes, even survival. This work provided a framework for analyzing the regulation of dosage effect, which will shed a light on understanding the oncogenic and tumor suppressive mechanisms of CNA. Besides, new cancer-related miRNAs were identified.

## 

Activation of oncogenes and inactivation of tumor suppressor genes are key mechanisms for tumor development. In this decade, researchers found copy number alteration (CNA) can induce gene expression changes through dosage effect, therefore result in specific phenotypes and diseases^1^,^2^,^3^. Driving mutations might leave a genomic “footprint”, so aberrant expressed genes with CNA are more likely to be important genes that drive the expression patterns of passenger gene module^4^. Most studies that integrate copy number and gene expression focus on genes whose expression is correlated with the copy number of the cognate DNA^4^,^5^,^6^, as candidate targets for cancer therapy. That is to say, genes with a copy number amplification and an up-regulation of expression were predicted as oncogenes, genes with a copy number deletion and a down-regulation of expression were predicted as tumor suppressor genes. For instance, the upregulated expression of MYC in the 8q24.21 amplified region is commonly observed in cancer, so MYC was considered as an oncogene^7^. PTEN at deleted region 10q23.31 always have low expression, so it was considered as a tumor suppressor gene^8^. Subsequent experiments further proved the functional roles of MYC^9^ and PTEN^10^.

Interestingly, researchers found that the change in copy number doesn’t always result in a concordant change in expression. In other words, the dosage sensitivity differs in different samples. In 2008, Gu *et al.* pointed out that the global correlation between gene expression and copy number is consistent, but relatively weak^11^. In 2011, Huang *et al.* claimed that most genes are not subject to dosage effect due to the transcription regulatory mechanisms^2^. Another typical example is that Samur *et al.* recently exhibited the variation of gene dosage effect in two subtypes of multiple myeloma, and discussed the functional consequences of the divergence in dosage sensitivity^12^. Their work has proven that studies on gene dosage sensitivity are of great value to the cancer field. They proposed an assumption about the existence of a regulatory network from copy number to gene expression, but without a validation that integrates any regulators such as transcriptional factors, miRNAs or other non-coding RNAs. So, what is the reason that the transcription of mRNA won’t always subsequently change when copy number changes? Little is known about factors that may regulate the alteration of copy number, the complexity of the transcriptional regulation mechanisms in biological systems and the difficulty of integrative analysis of multi-omics data make it a great challenge to answer the question. With recent advances in microarray and sequencing technology, large databases like The Cancer Genome Atlas (TCGA^13^) Project and the Cancer Cell Line Encyclopedia (CCLE^14^) has contained a mass of high quality cancer datasets. The increasing availability of genomic, transcriptomic and proteomic data from the same sample has made integrative analysis based on computational methods an attractive research area^15^. In recent years, as the dataset sizes of multiple cancer types reach hundreds or thousands of samples, a great opportunity is provided for the systematic identification and analyzation of the mechanisms of CNA regulation.

miRNAs are essential post-transcriptional regulators of gene expression, many studies had proven that they’re involved in tumor progression^16^. Detailed studies over the past decade have significantly extended our knowledge of miRNA, including their expression, target genes and biological functions. The results of the transient transfection of DNA plasmids performed by Bleris *et al.* showed that miRNAs could regulate gene dosage effect^17^, Ebert and Sharp have also discussed how miRNAs can help in conferring robustness to biological processes^18^. It is worth noting that, most of the existing studies that use “miRNA” and “CNA” as keywords often aim to identify miRNAs whose expression changes are driven by CNAs within their own genomic regions^19^,^20^, but rarely focus on their influence on target genes within CNA regions, which we call CNA genes for short. However, Dvinge *et al.* analyzed 1302 breast tumor samples and revealed that miRNA expression has low consistency with its own CNA^21^. Therefore, analyzing the regulatory role of miRNAs on CNA genes will shed new light on understanding the mechanisms of cancer, and identifying new prognosis or therapy targets.

Here, we built a framework integrating mRNA expression, miRNA expression, copy number profiles and transcriptional regulatory relationships, to reveal the potential contributions of miRNAs to gene dosage sensitivity, and the consequence of these phenomena on oncogenesis and tumor suppression activities. We implement this framework on breast cancer, glioblastoma and ovarian cancer, and three main results are notable from this study: (1) The dosage sensitivity of genes in the three tumors are relatively weak. In fact, genes with the highest dosage sensitivity conformed to the expectations for dosage effect in only about half of the CNA samples. (2) Genes with high dosage sensitivity have more miRNA binding sites and are targeted by more miRNAs. (3) We also scored miRNAs based on their impact on amplified/deleted genes. miRNAs like hsa-let-7b-5p and hsa-miR-92a-3p were found to have significant influence on gene dosage sensitivity, the results of functional analysis and survival analysis indicated that they may be potential prognosis or therapy targets.

## Materials and Methods

### Data

Gene expression (GE) and miRNA expression data of same cancer samples were downloaded from TCGA^13^, corresponding copy number (CN) data were obtained from Ciriello *et al.*^22^. The number of the samples with CN and GE in breast cancer (BRCA), glioblastoma (GBM) and ovarian cancer (OV) is 505, 552 and 562, respectively. When miRNA expression data were considered, the numbers decrease to 310, 541 and 558, respectively. Notably, to include as many samples and to make the results more reliable, samples with CN and GE were used when analyzing the gene dosage sensitivity, while samples with three types of data were used when identifying miRNAs.

Predicted miR–target gene relation were downloaded from TargetScan^23^, validated targets were obtained from miRecord^24^ and Tarbase^25^. Putative miRNA binding sites were obtained from UCSC^26^. The set of cancer-related genes were downloaded from the Cancer Gene Census of COSMIC^27^ [http://cancer.sanger.ac.uk/cancergenome/projects/cosmic/], including a list of 526 genes known to be involved in cancer. Protein-protein interaction (PPI) data were downloaded from HPRD^28^, containing 39239 interactions among 9865 genes. We acquired the list of 145 essential genes and a larger list of 332 genes from the study of Davoli *et al.*^29^, which integrated the KEGG database, the housekeeping genes and genes with high evolutionary conservation. We used the 1591 genes from DrugBank as a source of cancer therapeutic targets. Human protein complex data were obtained from CORUM database^30^, which lists 2542 genes encoding protein complexes.

### Discretization of copy number and gene expression data

**Figure 1:**
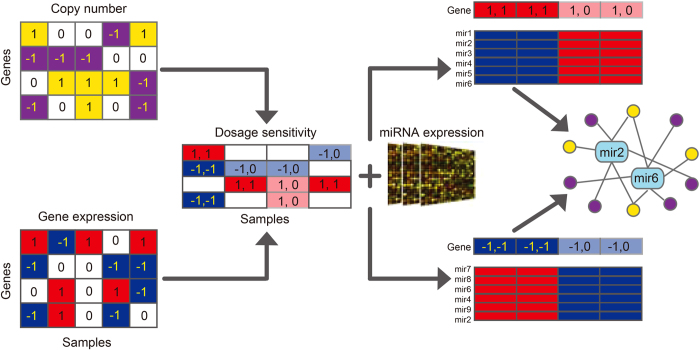
Framework of the evaluation of dosage sensitivity and the identification of miRNAs that modulate dosage response. In the copy number matrix, yellow represents amplification, purple represents deletion. In the expression matrix of genes and miRNAs, red represents upregulation, blue represents downregulation. In the dosage sensitivity matrix, blue and red represents dosage sensitive samples with copy number deletion and amplification, respectively; and light blue and red represents dosage resistant samples with copy number deletion and amplification, respectively.

We acquired discrete copy number data from Ciriello *et al.*^22^ (Fig. 1). In the copy number profile, 1 represents an amplification of the gene, −1 represents a deletion, and 0 represents the normal diploid. We calculated the mean expression value and standard deviation (SD) of each gene across all samples. Gene expression values were divided into discrete levels, the values that are higher than mean + SD were set to 1, the values that are lower than mean-SD were set to −1, other values were set to 0. In this discrete expression matrix, 1 represents high expression, −1 represents low expression, and 0 is intermediate.

### Gene dosage sensitivity and its feature analysis

Based on discrete matrices of CN and gene GE, we obtained a matrix of gene dosage sensitivity (Fig. 1). The three matrices have the same genes and samples as rows and columns. Each item in the matrix indicates two distinct factors, the first number denotes the state of CN, and the second number denotes the state of GE. For each gene, samples with state (1,1) and (−1,−1) were considered to be dosage sensitive, the proportion of dosage sensitive samples in all CNA samples was taken as a measure for the dosage sensitivity of that gene. We then constructed two groups: high dosage sensitive genes were defined as the top 500 genes ranked by the proportion of dosage sensitive samples, and low dosage sensitive genes was defined as the bottom 500 genes.

Between the two groups, we compared the number of miRNA binding sites, the number of miRNAs targeting them, and the node degrees in PPI network. The wilcoxcon rank-sum test was used to determine the statistical significance.

Gene set enrichment analysis using feature sets was performed on high/low dosage sensitive genesets, separately. Cumulative hypergeometric test was used in the analysis.
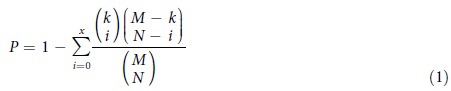


In the formula above, *x* represents the number of overlap genes between high/low dosage sensitive genesets and each feature sets. *M* is the total number of human genes, *N* is the number of the genes filtered out by us, so *N* = 500. *K* is the number of genes in feature sets.

### Identifying miRNAs that intervene gene dosage sensitivity by integrated approach

For each gene *k*, all samples with its CNA were divided into two groups: dosage sensitive group *G^s^_k_*^,^ and dosage resistant group *G^r k^*. In detail, amplified genes were divided into (1,1) group and (1,0) group, deleted genes were divided into (−1,−1) and (−1,0) group (See Fig. 1 and Supplementary Fig. S1). If one of the two groups had less than 10% samples, then this gene was excluded for a credible result in further analysis. For genes with both amplified samples and deleted samples at the same time, samples were divided into four groups: (1,1), (1,0), (−1,−1), (−1,0). This kind of gene should be excluded if and only if the dosage sensitive (resistant) samples were less than 10% in both amplified and deleted samples. Some genes had (1,−1) or (−1,1) samples, which occurred in less than 10% of total CNA samples. These samples were removed as they are likely to be the result of experimental errors, even though they have some biological significance, the number of samples remained too small to allow a valid statistical analysis. After that, based on the sample classification for genes, we grouped the samples for miRNAs targeting these genes.

The formula shown below was used to calculate amplified gene *k*:


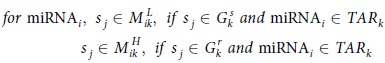


The formula shown below was used to calculate deleted gene*k*:


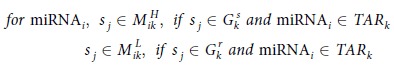


In the formula, *TAR*_*k*_ represents the set of miRNAs targeting *k*, *s*_*j*_ represents *j*th sample. Samples of *miRNAi* were divided into *M^L^_ik_* and *M^H^*_ik_ based on the dosage sensitivity of *k*. Then we evaluated whether *miRNAi*’s expression is significantly lower in *M^L^*_ik_ than *M^H^_ik_* using single-tailed t test, and the p value was recorded as P_ik_. *miRNAi*-gene*k* pairs with a P_ik_ < 0.05 were retained as significant.

To evaluate the significance of miRNA-gene pairs, we generated 1,000 independent datasets by random permutation, in which the samples were randomly reshuffled while preserving both the number of dosage sensitive samples and dosage resistant samples. In the sample permutation test, the Q-value_sample_ of each miRNA-gene pair is defined as the proportion of pairs with a higher real p-value. miRNA-gene pairs with Q-value_sample_ > 0.1 were excluded. Besides, in order to reduce the influence of false positives in miRNA target data and to ensure the biological significance of miRNAs, we also screened the miRNA-gene relationship pairs by permutation tests. For each gene, we randomly chose 100 miRNAs (with replacement) and computed single-tailed t-statistics using the method given above. Q-value_relationship_ for each miRNA-gene pair is defined as the proportion of pairs with higher real P_ik_ compared with random ones, miRNA-gene pairs with Q-value_relationship_ > 0.1 were excluded.

### Scoring miRNAs based on their influence on dosage sensitivity

One miRNA could affect the dosage sensitivity of multiple genes. Therefore, with miRNA-gene pairs from single-tailed t test, we scored each miRNA using the formula below:


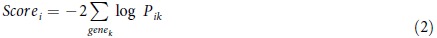


In which *gene*_*k*_ represents the target gene whose dosage sensitivity was regulated by *miRNAi*. To investigate both the tumor-suppressive and tumor-promoting roles of miRNAs, the ability of miRNAs regulating amplified genes or deleted genes were scored separately.

### Survival analysis

All survival analyses in this study are cox regression analyses. Age and tumor stage are employed as covariates.

## Results

### Gene dosage sensitivity is heterogeneous in different tumor samples

By integrating paired copy number and gene expression profiles of BRCA, GBM and OV cancer samples from TCGA, we obtained a whole genome dosage sensitivity matrix for each tumor, as shown in Fig. 1. Each term in the matrix is a length-2 vector, (1,1) represents a copy number gain and upregulation, (−1,−1) represents a loss and downregulation. Samples with a (1,1) or (−1,−1) were considered to be dosage sensitive, and samples with a (1,0) or (−1,0) were considered to be dosage resistant. We found that, for the samples with CNA of the same gene, the corresponding expression changes were observed only in some samples, indicating the heterogeneity of gene dosage sensitivity in different tumor samples.

We calculated the proportion of dosage sensitive samples for each gene. According to our results, DIABLO has the highest proportion (50.8%) in BRCA, KRR1 has the highest proportion (50.9%) in GBM, and SNRPF has the highest proportion (32.4%) in OV. Among all these genes with CNA, 76 genes were found to have a proportion higher than 40% in BRCA, while 174 were found in GBM. Only 12 genes reached a proportion of 30% in OV (Supplementary Fig. S2). Obviously, BRCA and GBM are more responsive to CNA than OV. Ciriello *et al.* discussed that GBM is developing mostly based on mutations, while OV is based on CNAs^22^. This result does not contradict our observation, as the conclusion of Ciriello *et al.* was based on few hundreds of genomic mutations that were recurrent in multiple cancers, rather than focusing on the status of corresponding expression change of CNA genes at the whole-genome scale. In our results, 1332 and 2223 genes had a CNA rate over 60% in GBM and BRCA respectively, but for OV, the number is an astounding 9677. These numbers indicated that recurrent CNAs are more common in OV, which is consistent with the conclusion of Ciriello *et al.* However, even with the huge amount of CNAs, the proportion of samples with expressional and functional alterations in OV was less than which in BRCA and GBM. These findings indicated that genes with CNA are not always differentially expressed in tumor. Samur *et al.*'s study of multiple myeloma also had the similar result, suggesting the diversity of gene dosage sensitivity in different samples may be due to specific regulatory factors.

### Genes with higher dosage sensitivity are targeted by more miRNAs

Since dosage sensitive genes are likely to be drivers of oncogenesis, it would be helpful to analyze the relationship of gene dosage sensitivity and its regulatory pattern in order to find potential regulatory factors.

**Figure 2:**
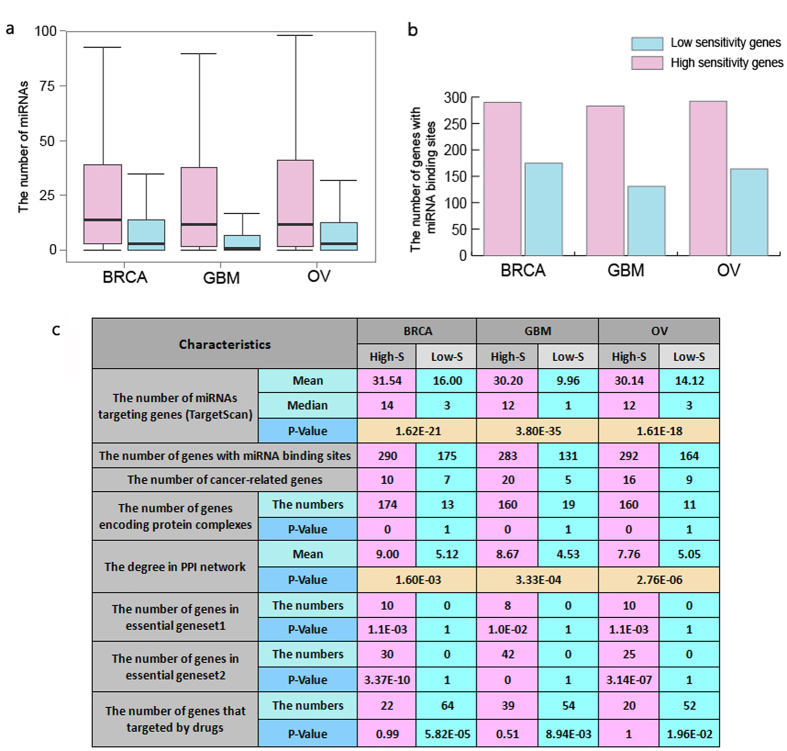
Comparison between the features of high sensitivity genes and low sensitivity genes. **a**: High sensitivity genes are regulated by more miRNAs.(single-tailed t test, results see Fig. 2c).**b**: High sensitivity genes have more miRNA binding sites. **c**: Details of the comparison results for the features of high sensitivity genes and low sensitivity genes. High-S and Low-S denotes for the set of high and low sensitivity genes, respectively. The P-value of Wilcoxon rank sum test was used to measure the differences between High-S and Low-S in the number of miRNAs and the degree in PPI network. The other P-values were calculated with the cumulative hypergeometric test.

We compared genes with high and low dosage sensitivity by the amount of miRNAs targeting them and the amount of miRNA binding sites in them based on the predicted miRNA-target in TargetScan and miRNA binding information in UCSC. As shown in Fig. 2, genes with high dosage sensitivity were targeted by more miRNAs (Fig. 2a), and had more miRNA binding sites (Fig. 2b). To deal with the high false positive rate of predicted miRNA targets and to ensure the correctness of our conclusions, we also investigated the dosage sensitivity of genes in the validated miRNA-gene pairs. The results showed that, in BRCA, GBM and OV, 58, 42 and 51 high dosage-sensitivity genes (high sensitivity genes for short) are confirmed to be regulated, but the numbers sharply reduced to 14, 2 and 7 when it comes to low dosage-sensitivity genes(low sensitivity genes for short). This indicated that high sensitivity genes (or driver genes) are under complicated and subtle post-transcriptional regulation. To determine whether the dosage sensitivity is correlated with gene length and 3’UTR length, we calculated the Pearson correlation coefficient (PCC) between them. The correlation between gene dosage sensitivity and gene length are similar in three types of cancers (BRCA: -0.052, GBM: -0.0056, OV: -0.022). And the correlation between gene dosage sensitivity and 3’UTR length were also very weak (BRCA: -0.013, GBM: -0.0079, OV: -0.0043). This indicates that the tendency of high sensitivity genes to be regulated by miRNAs is not due to their gene length or even 3’UTR length, but maybe more complicated functional and evolutionary reasons. Furthermore, we calculated the correlation between gene dosage sensitivity and mean of expression, the result showed that gene dosage sensitivity was not dependent on gene expression level. The correlations in three types of cancer were 0.045, -0.020 and 0.0021, respectively. In addition, we also found that the high dosage sensitivity genes in three types of cancer tend to have specific characteristics compared with low sensitivity genes (See Fig. 2c, Supplementary Fig. S3), suggesting their specific functional roles. In detail, high sensitivity genes (1) contained more cancer-related genes; (2) enriched to the geneset of genes encoding protein complexes; (3) exhibited higher connectivity in the PPI network; (4) enriched to essential genes; (5) depleted in drug target geneset, while low sensitivity genes were enriched to drug targets. That may be due to that many essential genes are in the high sensitivity group, the drugs targeting these genes can harm normal cells as well as cancer cells.

All these results suggest that gene dosage sensitivity implies underlying mechanisms of tumorigenesis and malignant progression, and should be of value in cancer research. However, even the most sensitive gene didn’t show a corresponding phenotype in about half of the samples with CNA. Uncovering the modulators of dosage sensitivity will help dissect the patterns of CNA affecting cancer progress.

### miRNA expression could serve as a trigger or balancer in cancer by affecting the dosage sensitivity of target genes

By the integration of mRNA expression, miRNA expression, copy number profiles of three types of cancer, the sample classification method (See Methods) and the permutation test, miRNAs significantly regulating the dosage sensitivity of amplified/deleted genes were identified.

**Figure 3:**
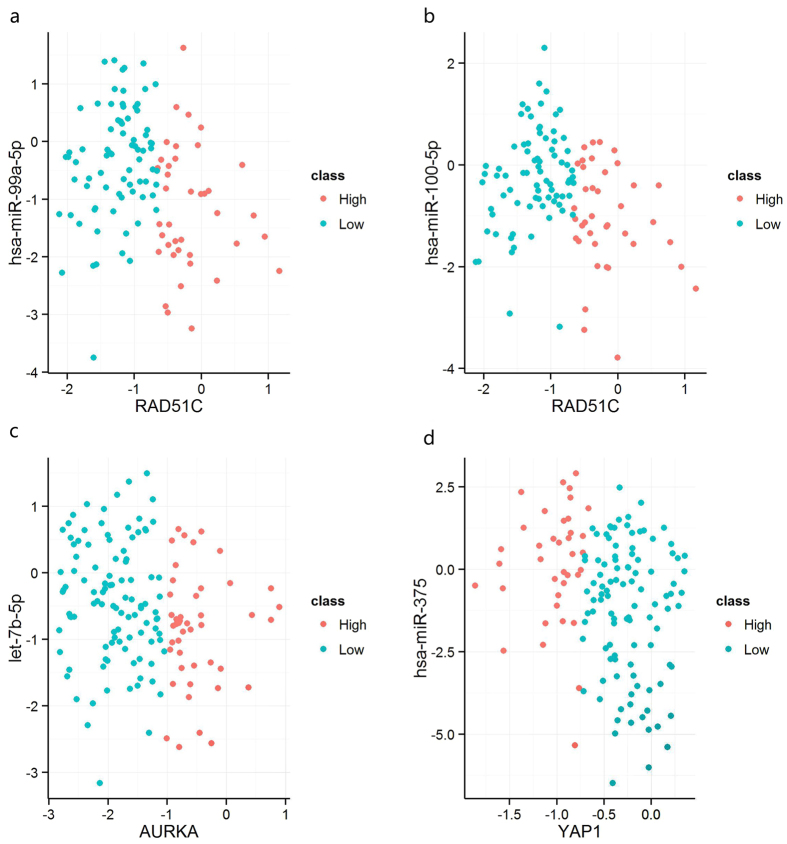
miRNAs expressions relate to the dosage sensitivity of oncogenes/tumor suppressor genes in BRCA. **a**, **b**: The dosage sensitivity of amplified oncogene RAD51C in BRCA is related to the expression of hsa-miR-99a-5p and hsa-miR-100-5p. **c**: The dosage sensitivity of amplified oncogene AURKA in BRCA is related to the expression of let-7b-5p. **d**: The dosage sensitivity of deleted tumor suppressor gene YAP1 in BRCA is related to the expression of hsa-miR-375.

**Figure 4:**
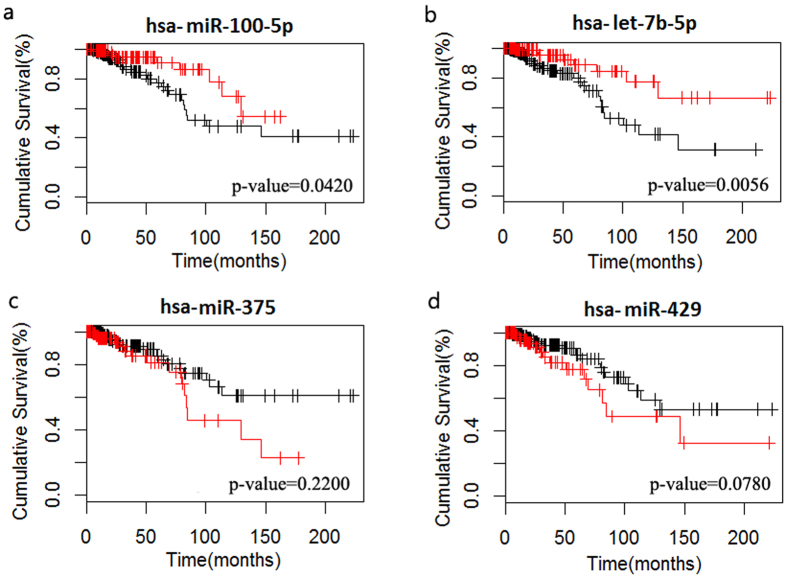
Survival analysis of miRNAs regulating the dosage sensitivity of amplified/deleted genes in BRCA. **a** and **b** indicate that samples with high expression of hsa-miR-100-5p and hsa-let-7b-5p that regulating the dosage sensitivities of amplified genes had a longer survival time. **c** and **d** indicate that samples with low expression of hsa-miR-375 and hsa-miR-429 regulating the dosage sensitivities of deleted genes had a longer survival time.

We found that, the dosage sensitivity of many amplified genes is significantly related to the expression of some of miRNAs targeting them. For example, RAD51C at 17q23 cooperate with BRCA1, BRCA2 in DNA damage response pathway^31^,^32^, and is critical for G(2)/M checkpoint control^32^. RAD51C is repeatedly reported to be amplified in BRCA^33^,^34^ and involved in the tumor process as a driver oncogene of BRCA^33^,^34^. 117 in 310 samples (37.7%) showed RAD51C amplification, but only 39 amplified samples had increased expression. The expression of hsa-miR-100-5p and hsa-miR-99a-5p was significantly lower in dosage sensitive samples of RAD51C (single-tailed t-test p-value: 1.70E-5 and 4.10E-4, See Fig. 3a,b), leading to the decreased inhibition of oncogene RAD51C. In the dosage resistant samples of RAD51C, the expression of the two miRNAs were relatively higher, which counteracted the tendency of high expression of RAD51C. A poor survival was observed in samples with the low expression of hsa-miR-100-5p (Fig. 4a), may be due to the incomplete inhibition of the expression of RAD51C. The study of Gebeshuber *et al.* showed that hsa-miR-100-5p could inhibit breast tumorigenesis.48.7% samples (151/310) had shown AURKA amplification, in which 46 are dosage sensitive samples^35^. AURKA at 20q13 can encode aurora kinase A, which is an essential protein in microtubule formation and cell cycle. This gene was found to be associated with the subtyping and prognosis of breast cancer^36^,^37^. We found that hsa-let-7b-5p may contribute to preventing the up-regulation of amplified AURKA (single-tailed t-test p value: 1.2E-3, See Fig. 3c), and help stabilize the expression of AURKA in the dosage resistant samples. Therefore, the high expression of hsa-let-7b-5p is speculated to have a role in suppressing tumorigenesis, as a balancer for cancer. We also found that samples with an overexpression of hsa-let-7b-5p had significant longer overall survival (Fig. 4b). Previous studies have proved its involvement in regulating on ER-alpha signaling in the ER-positive breast cancer^38^, regulating the self renewal and tumorigenicity of breast cancer cells^39^ and repressing cell proliferation pathways^40^, indicating its tumor suppressive role.

The dosage sensitivity of many deleted genes is also significantly related to the expression of some of miRNAs targeting them. RAB11A can regulate epidermal growth factor, its aberrant expression could promote the proliferation and motility of cancer cells^41^,^42^. 94 in 310 samples (30.3%) showed RAB11A loss, in which 36 loss samples had decreased expression, the loss of RAB11A in other 58 samples didn’t induce any corresponding expression change. Two miRNAs were recognized to be related to the dosage sensitivity of RAB11A, namely hsa-miR-532-5p and hsa-miR-92a-3p (single-tailed t-test p value is 3.7E-3, 2.7E-3, respectively). Another example is YAP1, whose transcript is known to be the nuclear effector the Hippo signaling pathway, which plays an important role in development, growth, repair, and homeostasis. This gene is reported to be involved in the progression of multiple cancers^43^,^44^. Recently, Cottini *et al.* found that YAP1 can trigger DNA damage-induced apoptosis, thus inhibit the unlimited proliferation of liver cancer cells^45^. As a high deletion rate of YAP1 (45.5%, 141/310 samples) was observed in BRCA, we assume that YAP1 may also trigger apoptosis in breast cancer cells. A significant differential expression of hsa-miR-375 between the dosage sensitive and resistant samples of YAP1 is observed (p value: 1.2E-3, Fig. 3d). Therefore, the low expression of hsa-miR-375 could possibly associates with deleted YAP1 expression balance, thus inducing the apoptosis of cancer cells. On the contrary, the overexpression of hsa-miR-375 could contribute to inhibiting the expression of YAP1 that already had a copy number loss, may acting as a trigger for cancer. The results of survival analysis indicated that samples with the overexpression of hsa-mir-375 had shorter overall survival, suggesting its potential as a BRCA prognostic target. It has been also reported that hsa-mir-375 was related to YAP1 in lung cancer, and its activation was consider to be involved in cancer progression^46^.

As one miRNA could target multiple genes, we scored and ranked each miRNA based on their impacts on amplified/deleted genes (See Fig. 1, Supplementary Fig. S2 and S3). Top 10 miRNAs regulating amplified genes were identified (See Table 1), most of which were validated to be tumor suppressive, like hsa-let-7b-5p^40^ and hsa-miR-142-3p^47^. In the top 10 miRNAs in BRCA, hsa-let-7b-5p and hsa-miR-100-5p were found to be prognostic factors for BRCA (See Fig. 4). Most of the top 10 miRNAs regulating deleted genes were proved to be oncogenic by previous studies(See Table 2), for example, hsa-miR-92a-3p^48^ and hsa-miR-19b^49^. We also observed poor survival in the BRCA samples with hsa-miR-429 overexpression (Fig. 4d). Besides, some CNA genes such as SLC12A4, RBL2, POU2F1, MCL1, ALDH9A1 were found to be targeted by several miRNAs, indicating that they maybe miRNA-sensitive CNA genes.

These results showed that, miRNAs have a potential role in promoting or suppressing tumorigenesis by mediating the dosage sensitivity of target genes. Identifying miRNAs affecting the dosage sensitivity of tumor suppressor genes and oncogenes would help us to find new prognostic factors and therapeutic targets.

### miRNAs could regulate oncogenic functions/pathways affected by CNA

**Figure 5:**
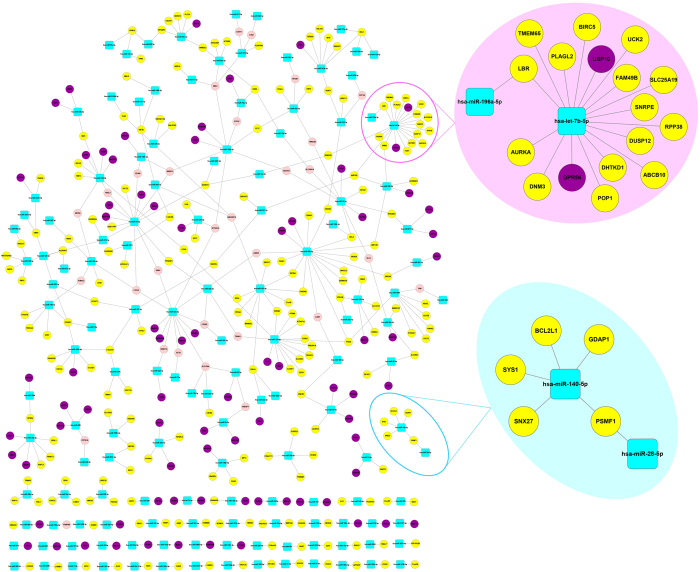
Dosage sensitivity regulation network in breast cancer. Blue rounded rectangle nodes denote for miRNA, yellow nodes denote for amplified genes, purple nodes denote for deleted genes, pink nodes denote for the genes which are amplified in some samples, and deleted in the other samples.

All these relationships between miRNA and gene dosage sensitivity could form a dosage sensitivity regulatory network (Fig. 5). The dosage sensitivity regulatory network of BRCA contains 140 miRNAs, 196 amplified genes, 78 deleted genes, and 30 ‘flexible’ genes that are amplified in some samples and deleted in the other samples. The GBM network contains 226 miRNAs, 179 amplified genes, 102 deleted genes, and 74 ‘flexible’ genes (Supplementary Fig. S4). The OV network contains 338 miRNAs, 283 amplified genes, 244 deleted genes, and 114 ‘flexible’ genes (Supplementary Fig. S5).

We uncovered distinct modules from the dosage sensitivity mediation network. For example, hsa-miR-140-5p and its five amplified targets have formed an individual module in the BRCA network together with hsa-miR-28-5p (Fig. 5). In the module, BCL2L1 at 20q11.21participate in the KEGG pathway of apoptosis^50^ and is involved in prevention of apoptosis^51^; PSMF1 can encode a proteasome inhibitor^52^ and is involved in cancer-related pathways like apoptosis, cell cycle checkpoints, DNA replication and activation of NF-kappaB in B cells^53^; GDAP1 is annotated to GO:0008219 (cell death). The amplification and upregulation of all three genes would lead to the disruption of normal apoptosis pathway in cells, thus promoting the development of tumor. In this study, hsa-miR-140-5p and hsa-miR-28-5p were predicted to counteract the high expression tendency of these amplified genes in the apoptosis pathway, suggesting their potential tumor suppressive role. Intriguingly, hsa-miR-140-5p has already been proven to suppress the growth, migration and invasion of cancer cells^54^,^55^.

In addition, a largest component containing most miRNAs and genes were identified in the BRCA network. In the network, hsa-let-7b-5p had the highest connectivity, 15 amplified genes and 2 deleted genes were under its regulation, including amplified AURKA (mentioned above), SNRPE in the spliceosome pathway, BIRC5 involved in the cancer pathways, and deleted USP10 which is involved in the MAPK signaling pathway.

We found that a lot of miRNAs mediate both amplified genes and deleted genes (Fig. 5), which provided a challenge to study the functional role of miRNAs. Since the fact that some CNAs are just passenger events during cancer development, we could speculate that reasonably, whether a specific miRNA is a trigger or a balancer is determined by whether its target is a driver. Besides, the function of a miRNA should be under the joint effect of the functions of its target genes. For instance, hsa-let-7b-5p is observed as tumor suppressive, the reason could be either or both: The targets of hsa-let-7b-5p included well-known oncogenes AURKA and BIRC5, or the targets involved much more amplified genes.

A lot of existing studies aim to identify miRNAs whose expression changes are driven by CNAs with in miRNAs’ genomic regions. Our work also showed that, miRNAs can affect the process of cancer by regulating their target CNA genes, but their own CNA levels may contribute to affecting the process of cancer as well. However the latter events are fewer. Using data of 505 BRCA samples, 552 GBM samples and 562 OV samples with both copy number and miRNA expression, R package DRI was implemented to identify miRNAs with concordant copy number/expression relationship. The R package DRI^56^ was based on statistical correlation test (three options are provided: Pearson’s correlation, Spearman’s rank correlation, modified Student’s t-test, and we used the Pearson’s correlation).All parameters were default. Only 28 miRNAs whose FDR was <0.05 and pearson correlation coefficient (PCC) between copy number and expression were greater than 0.4 in BRCA. Results for GBM and OV are better (97 and 122 miRNAs, respectively), but still much less than the miRNAs regulating amplified/deleted genes. The poor dependence of miRNAs’ expression on their own CNAs in BRCA was also reported by Dvinge *et al.*^21^.

## Discussion

CNA had been considered as a benign mutation in early studies. In recent years, more and more researchers have found that CNAs contribute to tumor progression^57^,^58^. Our study presented that the functional consequence of CNA in different cases cannot be roughly judged. Only in dosage sensitive samples could CNA induce tumorigenesis due to the expression change of amplified or deleted genes. Therefore, whether CNA is a benign event depends on factors that modulate the transcriptional progress of CNA genes.

In this work, we found that the expression of miRNAs can affect the dosage sensitivity of CNA genes. This result will provide a new insight into personalized medicine for cancer. Because with the unique CNA gene profile for a certain sample, controlling the expression level of specific miRNAs will balance the overexpression tendency of amplified oncogenes, or the low expression tendency of deleted tumor suppressor genes, thus inhibit cancer progression. So, this is a potential direction to correct CNA’s functional consequences by modifying miRNA level, but we admit that a lot of experimental validations are needed to propose a safe therapeutic strategy. In addition, the results of our work showed that, the dosage sensitive genes were more often targeted by miRNAs. This might be due to the functional roles of these genes, as they are likely to be essential genes and hubs in the biological networks, which are involved in processes that are under strict control of miRNAs. On the other hand, the regulation of miRNAs on dosage resistant genes (genes which are not dosage sensitive in most samples) are not as much. Other factors like DNA methylation, transcription factors, long non-coding RNAs and histone modifications may also have potential effects in disturbing the correlation between mRNA expression and CNA copy number. This paper provided a framework for studying the regulatory mechanisms of dosage sensitivity, with the development of technology and data collection, the regulatory mechanisms of gene dosage sensitivity would be clearer, promoting the prognosis and therapy of cancer.

In the field of transcriptional regulation, a common theme is the regulation from one type of functional molecular to another. Sample classification is an effective way to address this question. In this method, samples are categorized according to the features of one type of molecular, and regulatory relationships are then inferred by comparing the alteration of another factor between different categories. This idea is first suggested in Wang *et al.*'s study of identifying modulators of transcription factor activity^59^. The study of Akavia *et al.*^4^ categorized the samples based on the expression of driver genes, and identified the passenger modules altered by the driver. Sumazin *et al.* uncovered a miRNA-mediated network of RNA-RNA interactions^60^. Based on the assumption that miRNA could intervene the co-expression of genes, Dvinge *et al.* classified the samples by miRNA expression, and investigated whether there is difference in the co-expression of genes in different groups, and thus identified the miRNAs regulating gene co-expression^21^. In our work, samples are divided into two groups according to the dosage sensitivity of genes located in CNA region. We analyzed the differential miRNA expression between the two groups, and revealed miRNAs that regulate the functional consequences of CNA. Obviously, the sample classification method is applicable in identifying modulators. However, this method somewhat depends on the quantity of samples. Theoretically, the correctness of the results will increase with the amount of samples used. Ciriello *et al.* used five discrete copy number calls derived from the GISTIC algorithm, namely2, homozygous deletion; 1, heterozygous loss; 0, diploid; 1, one copy gain; 2, high-level amplification or multiple-copy gain. As the main focus of this work is to identify the miRNAs which can regulate CNA genes, we combined the homozygous deletion and heterozygous deletion into one group designated as “deletion”, as well as one copy gain and multiple-copy gain into another group designated as “amplification” to achieve reasonable sub-sample sizes. The development of public databases will bring a result more close to biological truth, and enable the use of this method in the studies of pan-cancer or other cancers.

The results of our method are also affected by the miRNA-target data. Experimental validated miRNA-target data is recommended, but there is currently not enough data to support the study. In the absence of experimental validated miRNA-target data, accurate whole-genome analyzation cannot be performed. Existing prediction approaches have their individual merits and demerits^61^,^62^, we chose TargetScan as the source of miRNA-target data, since the experiments of miRNA knockout and transfection showed its good performance^63^,^64^. However, because no golden standard for miRNA-CNA gene pairs at present, there is no direct way to give an overall false positive or negative assessment of identified regulation relationships. We performed permutation tests while identifying miRNAs regulating gene dosage sensitivity, in order to reduce the influence of false positives in the data. And some of relationships were validated by published literatures. False negatives in the predicted data may also lead to inaccuracy as actual targets are missed. We hope that we could perform a more accurate analysis by experimentally validating the regulatory relationships in the future.

Besides, specific CNA patterns were always used to distinguish cancer subtypes^65^,^66^, we did not consider cancer subtypes due to the incomplete data. We hope to examine the impacts of miRNA on dosage sensitivity for different cancer subtypes, and to identify subtype-specific miRNA modulators in the future.

## Conclusions

By integrating copy number profile, gene and miRNA expression, and transcriptional regulatory relationships, we developed an approach to identify tumor related miRNAs based on gene dosage sensitivity, and revealed miRNAs’ regulation effect on their amplified/deleted target genes. Our results suggested that the driver genes have a more subtle and complicated pattern of miRNA regulation, and the expression change of miRNAs could influence the dosage sensitivity of oncogenes and tumor suppressor genes, serving as a trigger or balancer in cancer. This work will provide new candidate targets for miRNA-based therapy and prognosis of cancer, and facilitate the further improvement of personalized medicine.

## Additional Information

**How to cite this article**: Li, K. *et al.* An integrated approach to reveal miRNAs' impacts on the functional consequence of copy number alterations in cancer. *Sci. Rep.* **5**, 11567; doi: 10.1038/srep11567 (2015).

## Supplementary Material

Supplementary Information

## Figures and Tables

**Table 1:** Top 10 miRNAs regulating amplified genes in BRCA.

R^a^	miRNAs	Amplified target genes	N^b^	Score	PMID^c^
1	hsa-let-7b-5p	AURKA,UCK2,BIRC5,RPP38,DNM3,DHTKD1,ANP32E,TMEM65,RBM12B,SNRPE,FAM49B,DUSP12,LBR,SLC25A19,POP1,PLAGL2,ABCB10	17	372.01	17699775 20133835
2	hsa-miR-590-3p	DENND1B,PARD6B,PDZK1,TPD52,VPS13B,ANGEL2,ERLIN2,ZBTB41,ZNF238,TSNAX,HELZ,WDR26,RNF139	13	363.00	
3	hsa-miR-155-5p	VEZF1,CLUAP1,TPD52,ATP6V1H,EDEM3,PRKAR1A,TROVE2,PFDN4,SLC30A1,COG2,CCND1	11	241.07	
4	hsa-miR-142-3p	MANBAL,C1orf9,SDC4,PPFIA1,ZBTB41,ZNF217,EDEM3,TRPS1,PARD6B	9	218.57	23619912 21482222
5	hsa-miR-140-5p	SYS1,BCL2L1,GDAP1,PSMF1,SNX27	5	107.31	24039995
6	hsa-miR-186-5p	COG2,RNF2,EEF1D,NID1	4	105.83	23204228
7	hsa-miR-320b	ASH2L,TPD52,YWHAZ,ST3GAL1,CDC73,KCTD2	6	105.49	23705859
8	hsa-miR-193b-3p	RASSF5,TSEN34,MCL1,MTR,RSL1D1,SOAT1	6	104.91	
9	hsa-miR-100-5p	MAFG,SMG1,RAD51C,ALDH9A1,HIST2H2AA3	5	96.24	19396866
10	hsa-miR-450b-5p	C16orf72,ERCC4,C1orf21,KIAA0556,POU2F1	5	89.56	22266852

^a^The rank of the score of the miRNA.

^b^The amount of deleted genes targeted by the miRNA.

^c^The PubMed ID of an article in which the miRNA is reported.

**Table 2:** Top 10 miRNAs regulating deleted genes in BRCA.

R^a^	miRNAs	Deleted target genes	N^b^	Score	PMID^c^
1	hsa-miR-92a-3p	MED16,ASAH1,RNF123,SLC12A4,ZFYVE16,RIC8A,FYCO1,RAB11A,DCTD,TMEM184B,CPEB2,UBR1	12	228.57	23052693 22772712
2	hsa-miR-93-5p	FYCO1,UBR1,FBXL3,AKAP11,HERPUD1,SMG6,CD164,DPP8,FBXL5	9	186.09	19671678
3	hsa-miR-106b-5p	RBL2,STX12,AKAP11,CES2,ZFYVE16,RHOBTB2,CDC37L1	7	154.80	18922889 22286770
4	hsa-miR-16-5p	GLT8D1,TNRC6B,DLC1,RPL13,SLC39A14,CDC37L1	6	100.16	
5	hsa-miR-186-5p	ITM2B,MRFAP1,FBXL3,ZBTB4,APEH	5	93.17	
6	hsa-miR-429	PPP2CA,RHOT1,UBE2B,TBC1D12	4	76.04	20514023
7	hsa-miR-590-3p	MTMR9,ASAH1,IL6ST,HDHD2	4	73.52	
8	hsa-miR-19b-3p	FYCO1,TOM1L2,ZBTB4	3	66.87	24831732
9	hsa-miR-25-3p	PDGFRL,SLC12A4,SREBF1	3	57.08	
10	hsa-miR-130b-3p	AKAP11,PAPD4,CD164	3	51.42	21112564

^a^The rank of the score of the miRNA.

^b^The amount of deleted genes targeted by the miRNA.

^c^The PubMed ID of an article in which the miRNA is reported.
