# Supplementary material for: An integrated approach to reveal miRNAs’ impacts on the functional consequence of copy number alterations in cancer
Source: Sci Rep. 2015 Jun 23;5:11567. doi: 10.1038/srep11567 (PMC4477324; doi:10.1038/srep11567)
Supplement: Supplementary Information [file srep11567-s1.pdf]

An integrated approach to reveal miRNAs' impacts on the functional consequence of copy number alterations in cancer

Kening Li[1, 2, §], Yongjing Liu[1, §],Yuanshuai Zhou[1], Rui Zhang[1], Ning Zhao[1], Zichuang Yan[1], Qiang Zhang[1], Shujuan Zhang[1], Fujun Qiu[1], Yan Xu[1,*]

[1]College of Bioinformatics Science and Technology, Harbin Medical University, Harbin 150081, China

[2]School of Life Sciences and Biotechnology, Shanghai Jiao Tong University, Shanghai 200240, China

[§]These authors contributed equally to this work

*Corresponding author

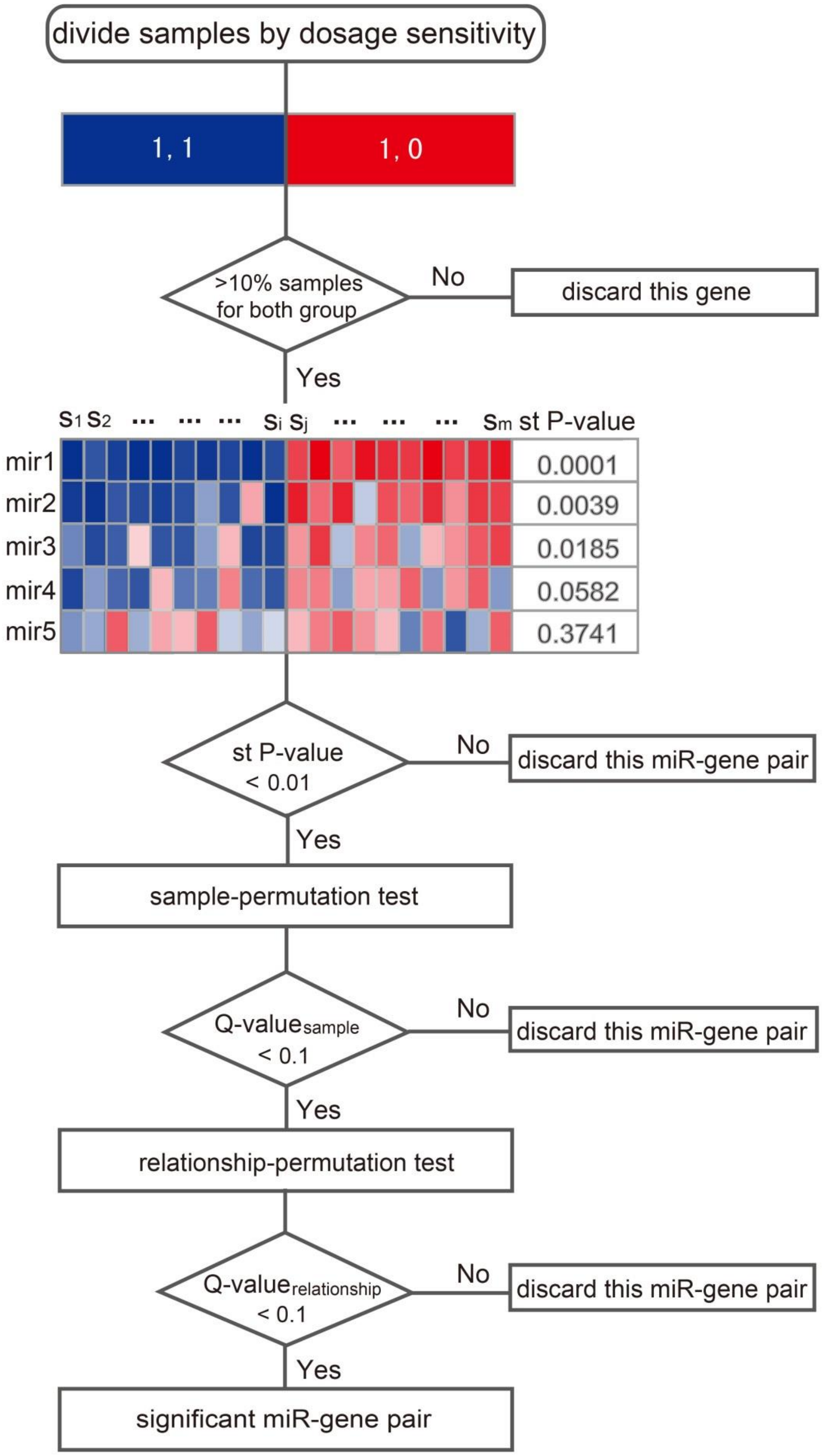

Supp. Figure S1. The flowchart for the identification of miRNAs that may intervene gene dosage sensitivity.

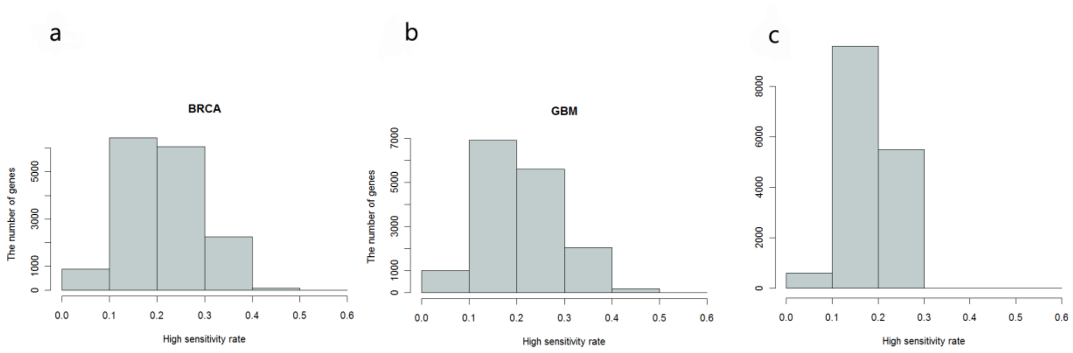

Supp. Figure S2. The high sensitivity rates in three types of cancer.

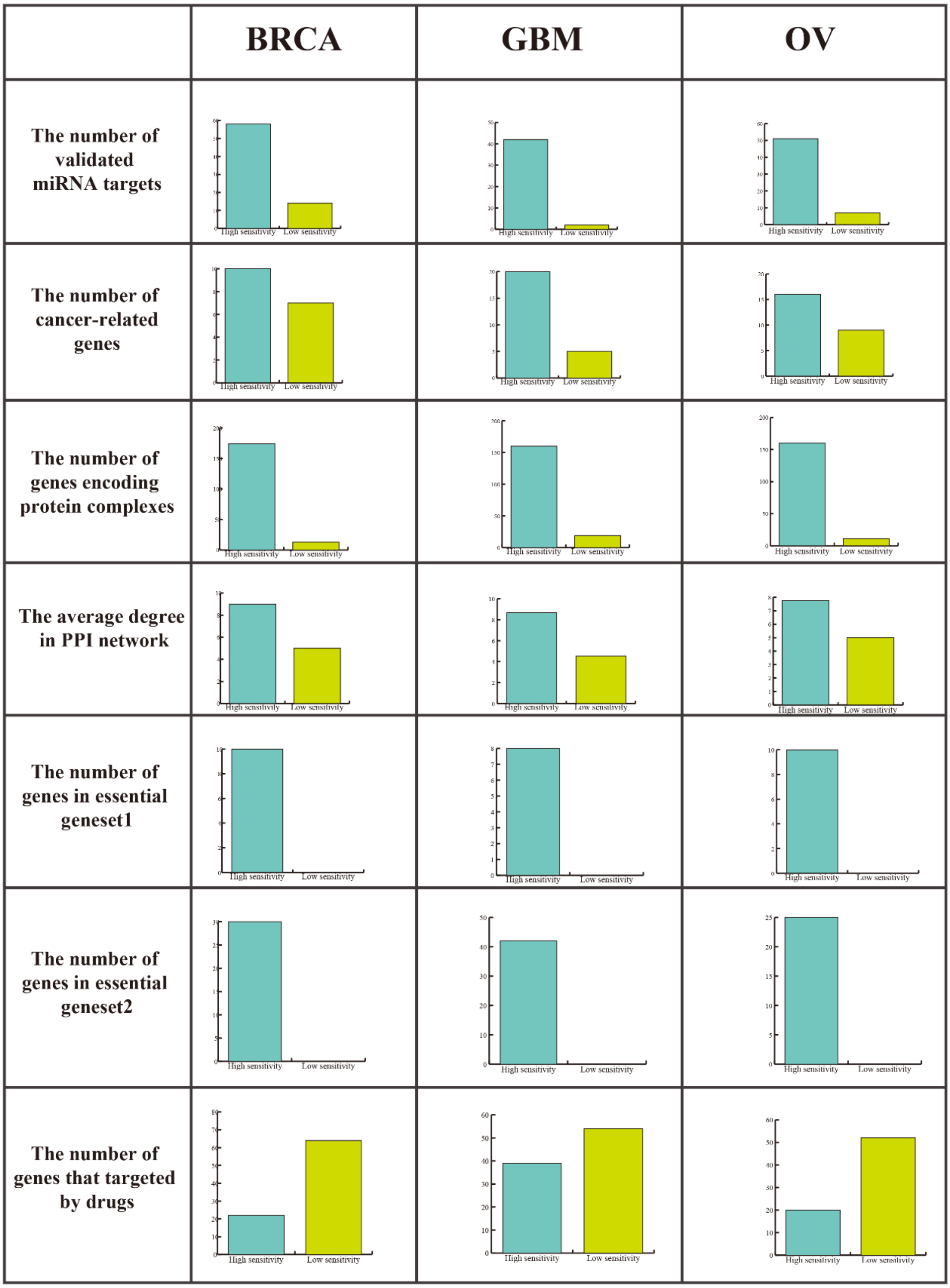

Supp. Figure S3. Comparison between high/low dosage sensitivity genes.

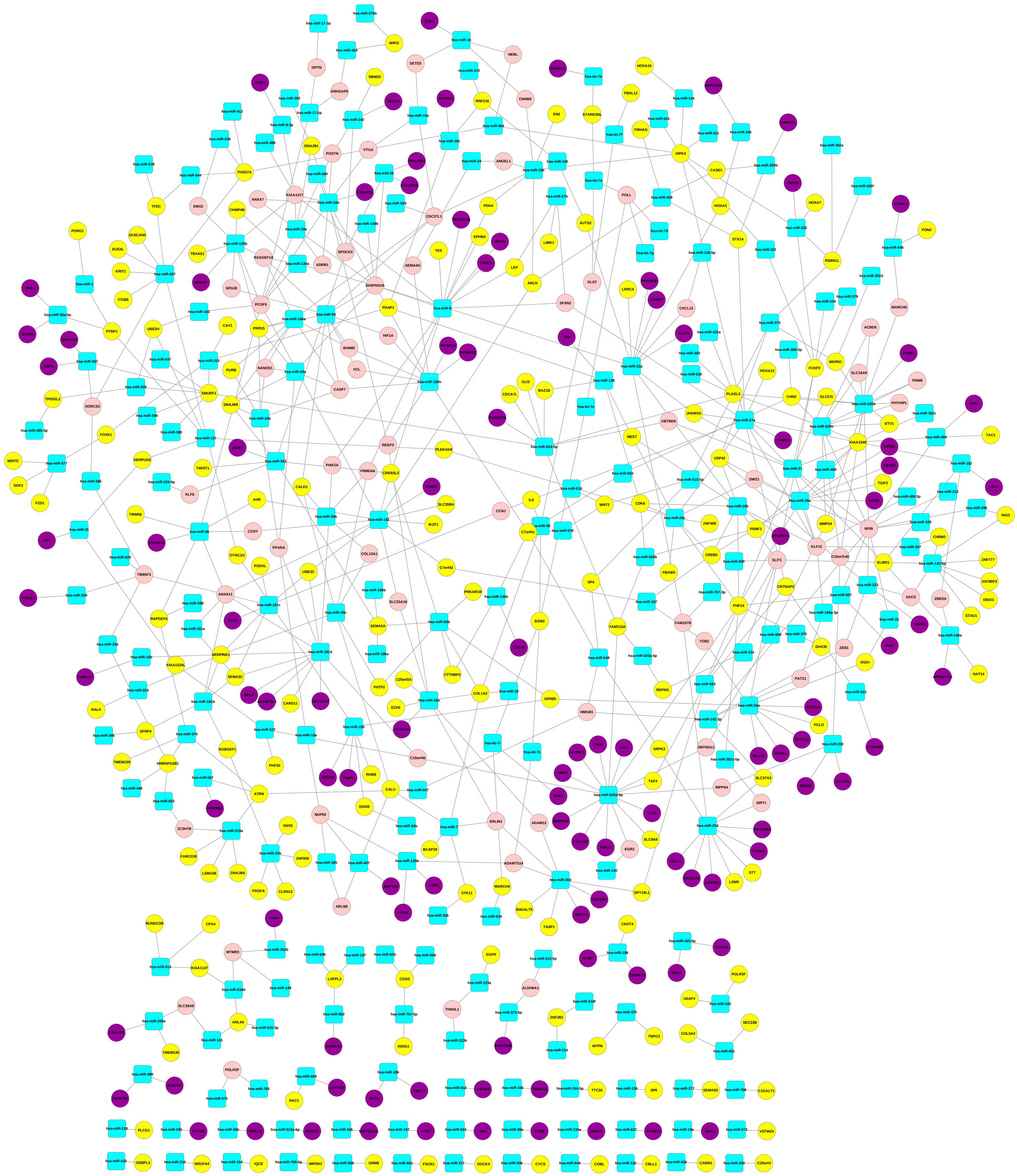

Supp. Figure S4. The dosage sensitivity regulation network of GBM. Blue nodes denote for miRNA, yellow nodes denote for amplified genes, purple nodes denote for deleted genes, pink nodes denote for the genes which are amplified in some samples, and deleted in the other samples.

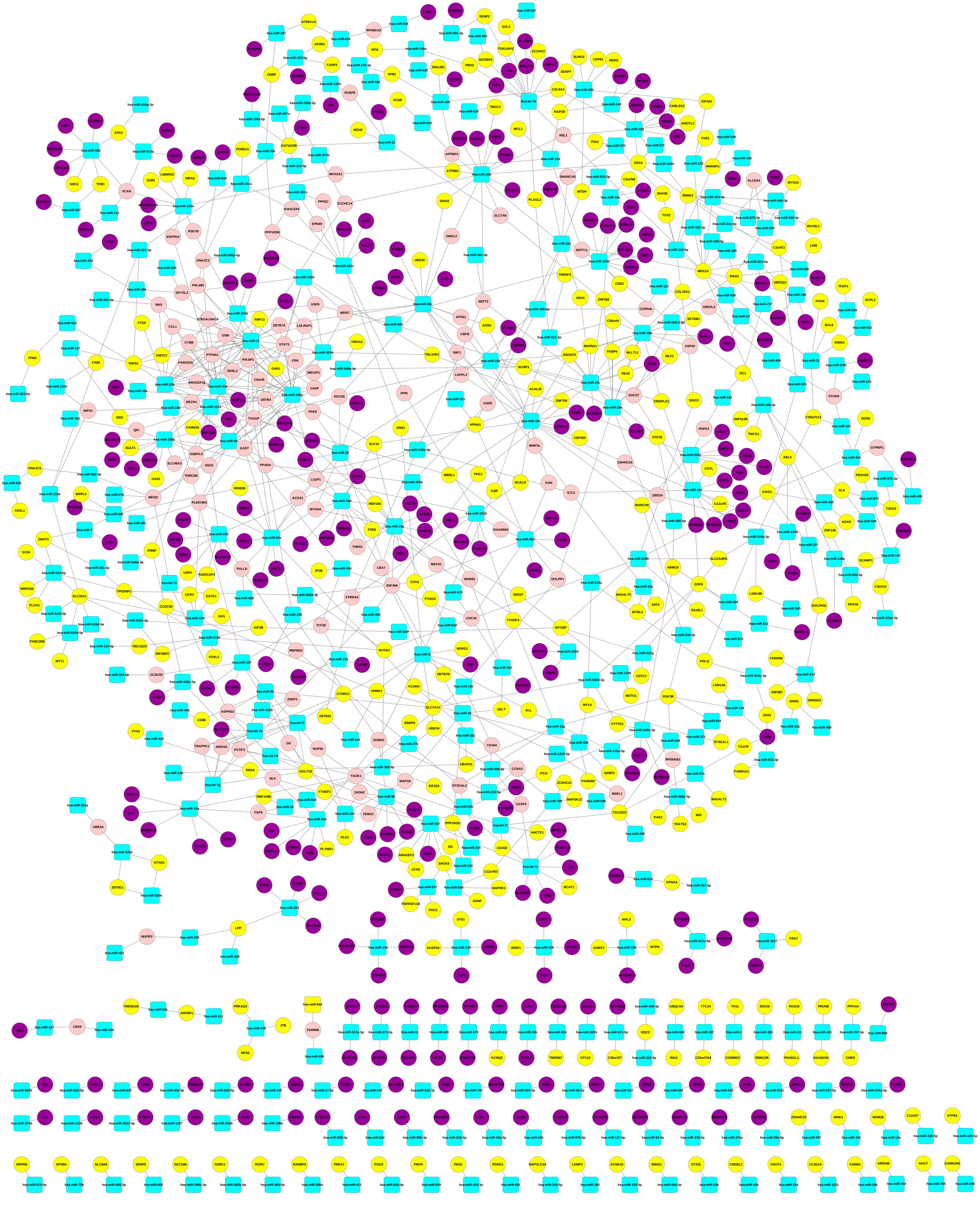

Supp. Figure S5. The dosage sensitivity regulation network of OV. Blue nodes denote for miRNA, yellow nodes denote for amplified genes, purple nodes denote for deleted genes, pink nodes denote for the genes which are amplified in some samples, and deleted in the other samples.

Supp. Table S1. Top 10 miRNAs regulating amplified genes in GBM

R[a]	miRNAs	Amplified target genes	N[b]	Score	PMID[c]
1	hsa-miR-27a	KIAA1549,ELMO1,USP42,JHDM1D,HOXA13,STX1A,CNTNAP2,HIPK2	8	195.13	19574223
2	hsa-miR-29c	USP42,MEST,ZNF498,PHF14,CDK6,CREB5,PLEKHA8,FBXW9	8	177.16	18390668
3	hsa-miR-181d	UBE3C,CARD11,SEMA3C,SERPINE1	4	142.42	
4	hsa-miR-182	UBE3C,AHR,CREB3L2,IKZF1,ANLN,C7orf42,SLC35B4	7	128.85	
5	hsa-miR-520b	ETV1,PLAGL2,MKRN1,CHN2,MMP24,KIAA1549	6	123.27	
6	hsa-miR-607	TFEC,ITGB8,DUS4L,DYNC1I1,ZKSCAN5,THSD7A,KRIT1	7	122.60	
7	hsa-miR-9	LEP,CREB3L2,TES,EPHB4,PDK4	5	121.86	23054677 21857646
8	hsa-miR-181c	SERPINE1,SEMA3C,UBE3C,PODXL	4	120.57	21895872
9	hsa-miR-2	JHDM1D,PHF14,FOXP2,KIAA1549,GLCCI1	5	116.44	
10	hsa-miR-92b	CTTNBP2,PRKAR1B,PHTF2,CREB3L2,SEMA3A,COL1A2,GPR85	7	114.44	

a: The rank of the score of the miRNA

b: The amount of deleted genes targeted by the miRNA

c: The PubMed ID of an article in which the miRNA is reported

Supp. Table S2. Top 10 miRNAs regulating deleted genes in GBM

R[a]	miRNAs	Deleted target genes	N[b]	Score	PMID[c]
1	hsa-miR-520d-5p	C10orf46,C10orf32,ZMYND11,INPP5A,NMT2,BMPR1A,H1F0,VTA1,CHUK,ATP5C1,EGR2,TNKS2	12	232.97	
2	hsa-miR-106b	KCNMA1,BAMBI,VCL,CASP7,HIF1A,REEP3,SH3PXD2A,PIP4K2A,SEMA4G	9	192.31	22893786<br>23302469
3	hsa-miR-93	SH3PXD2A,CDC37L1,SPG20,HIF1A,CASP7,VCL,BAMBI,RASGEF1A	8	180.08	23054677
4	hsa-miR-9	KIAA1217,SH3PXD2A,MYPN,THOC5,PI4K2A,FRMD4A,TBC1D22A,SFXN2	8	170.84	
5	hsa-miR-204	RDH11,SEC61A2,ADARB2,EIF4EBP2,FAM107B,SIRT1,PANK1	7	166.76	23204229
6	hsa-miR-520b	PHYHIPL,SLC16A9,MARCH5,C10orf140,ACBD5,TRIM8,CPEB3,NFIB	8	151.94	
7	hsa-miR-19b	PCGF5,POSTN,ANXA7,VTI1A,SH3PXD2A,KIAA1217,REEP3,ADRB1,C10orf26	9	143.35	23516263<br>23824915
8	hsa-miR-520e	TRIM8,SLC16A9,NFIB,C10orf140,ACBD5,PHYHIPL	6	122.40	
9	hsa-miR-23a	RUFY2,CXCL12,SLIT1,SH3PXD2A,PPP2R5E,FAM107B,CNNM1	7	119.45	23865473
10	hsa-miR-181d	RECK,CCDC117,ANXA11,PI4K2A,PPARA,C10orf46,ADAMTSL1	7	118.01	22570426<br>23054677

a: The rank of the score of the miRNA

b: The amount of deleted genes targeted by the miRNA

c: The PubMed ID of an article in which the miRNA is reported

Supp. Table S3. Top 10 miRNAs regulating amplified genes in OV

R[a]	miRNAs	Amplified target genes	N[b]	Score	PMID[c]
1	hsa-miR-29c	STK35,MLLT11,B3GNT5,CRISPLD1,SCHIP1,PEX5,ZNF282,PHC1,MAP6D1,KPNA1,FKBP4,SNX4	12	224.49	23904094 23728341
2	hsa-miR-29b	FKBP4,MLF1,COL22A1,ENAH,SETDB1,PEX5,C20orf4,SCHIP1,CRISPLD1,MLLT11,B3GNT5	11	190.40	24992675
3	hsa-let-7b	IGF2BP2,MEX3A,COL9A3,PLAGL2,SENP2,DVL3,PBX2,TOR1AIP2,ZCCHC3	9	174.78	24983365
4	hsa-miR-29a	FKBP4,MLF1,ZNF282,ARMC8,PEX5,SCHIP1,C20orf4,B3GNT5,MLLT11	9	169.78	23904094
5	hsa-miR-30e	MARCH6,AZIN1,CEP350,NCALD,ZNF706,KLHL20,KLF10,MBNL1,B3GNT5	9	165.52	23124598
6	hsa-miR-9	UBE2H,CTHRC1,IL6R,ELL,XRN1,NDRG1,UHMK1,ZBTB7B	8	162.56	22761433
7	hsa-miR-142-5p	DHX35,MEX3A,DIDO1,RBM12,TOX2,HNRNPU	6	123.47	22616694 22350417
8	hsa-miR-105	SLMO2,EIF4A2,SENP7,MTDH,CEP63,RAP2B,RERG	7	111.76	23950948
9	hsa-miR-188-3p	ZNF512B,TMCO1,SLA,ZNF146,ABL2,MEX3A	6	106.36	
10	hsa-miR-22	BCL9,C20orf112,RIMS4,POGK,MEX3A	5	104.06	20869762 22469921 21971665

a: The rank of the score of the miRNA

b: The amount of deleted genes targeted by the miRNA

c: The PubMed ID of an article in which the miRNA is reported

Supp. Table S4. Top 10 miRNAs regulating deleted genes in OV

R[a]	miRNAs	Deleted target genes	N[b]	Score	PMID[c]
1	hsa-miR-106a	LUZP1,TAGAP,LDLRAP1,PTP4A2,C9orf5,CYBB,PDLIM5,DPYSL2,ARHGEF18,CRK,ZBTB4,FAM134C,OSM,IL6ST,FAM102A,PLEKHM1,ST6GALNAC6,VASP,ZBTB7A,BECN1,SSH2,STAT3	22	413.92	24045973
2	hsa-miR-17	CCL1,TAGAP,LDLRAP1,DERL2,CRK,PDLIM5,ARHGEF18,CYBB,C9orf5,PTP4A2,NHS,FAM102A,IL6ST,OSM,ZBTB4,STAT3,BECN1,ZBTB7A,PPARA,VASP,ST6GALNAC6	21	383.48	
3	hsa-miR-20b	ZBTB4,OSM,IL6ST,FAM102A,ST6GALNAC6,VASP,ZBTB7A,BECN1,STAT3,DERL2,CCL1,TAGAP,NHS,PTP4A2,CYBB,ARHGEF18	16	298.61	24468585 22901144 21112772
4	hsa-miR-20a	CCL1,TAGAP,IL6ST,OSM,DERL2,WDFY2,ZBTB4,ENTPD4,ARHGEF18,PPP1R3B,CYBB,PPARA,ST6GALNAC6,PTP4A2,NHS	15	273.99	20458444 22449978 23390075
5	hsa-miR-30c	DMXL2,SBF1,ZBTB7A,PSMD7,WDR7,CBFB,LHFPL2,SOCS6,RFFL,STXBP5,BNIP3L,AP3S1,NOD2	13	229.79	22701724 23988701
6	hsa-miR-19a	CBX7,CDS1,PAK6,THBS1,ACTN1,MYO5A,MLLT6,ZNF469,SH3D19,MEF2C,ZBTB4	11	207.58	22465665
7	hsa-miR-106b	PPARA,OSBPL5,SOCS6,STAT3,RAD23B,BECN1,QKI,SLC46A3,CYLD,IL6ST,KIAA0513,SNX9	12	205.10	24641401
8	hsa-miR-101	ASPN,USP38,PCDH18,STC1,ZDHHC20,SOCS7,DOT1L,EFNB3,BMPR1B	9	185.04	21818714
9	hsa-miR-92a	WWP2,ACOX1,PALLD,CDC27,LUZP1,XYLT2,IL6ST,TAGAP,ATP8B1,MYO5A,PLEKHM1	11	180.89	19876917
10	hsa-miR-19b	TOR1B,CBX7,ZBTB4,LSM12,MEF2C,WDR45L,IL6ST,THBS1,PPP2R5E,MYO5A	10	176.21	24045973

a: The rank of the score of the miRNA

b: The amount of deleted genes targeted by the miRNA

c: The PubMed ID of an article in which the miRNA is reported
